# Current state of radiomic research in pancreatic cancer: focusing on study design and reproducibility of findings

**DOI:** 10.1007/s00330-023-09653-6

**Published:** 2023-04-20

**Authors:** James Alex Malcolm, Mark Tacey, Peter Gibbs, Belinda Lee, Hyun Soo Ko

**Affiliations:** 1 Faculty of Medicine, Dentistry and Health Sciences, The University of Melbourne, Melbourne, VIC Australia; 2 Department of Cancer Imaging, The Peter MacCallum Cancer Centre, Melbourne, VIC Australia; 3 Department of Biostatistics, Northern Health, Epping, VIC Australia; 4 Personalised Oncology Division, The Walter and Eliza Hall Institute of Medical Research, Melbourne, VIC Australia; 5 Department of Medical Oncology, Western Health, Melbourne, VIC Australia; 6 Department of Medical Oncology, Melbourne Health, Melbourne, VIC Australia; 7 Department of Medical Oncology, The Peter MacCallum Cancer Centre, Melbourne, VIC Australia; 8 Department of Medical Oncology, Northern Health, Epping, VIC Australia; 9 The Sir Peter MacCallum Department of Oncology, The University of Melbourne, Melbourne, VIC Australia; 10 Department of Diagnostic and Interventional Radiology, University Hospital Bonn, Venusberg-Campus 1, 53127 Bonn, Germany

**Keywords:** Pancreatic carcinoma, Biomarkers, Reproducibility of results, Precision medicine, Radiology

## Abstract

**Objectives:**

To critically appraise methodology and reproducibility of published studies on CT radiomics of pancreatic ductal adenocarcinoma (PDAC).

**Methods:**

PRISMA literature search of MEDLINE, PubMed, and Scopus databases was conducted from June to August 2022 relating to CT radiomics human research articles pertaining to PDAC diagnosis, treatment, and/ or prognosis, utilising Image Biomarker Standardisation Initiative-compliant (IBSI) radiomic software. Keyword search included [pancreas OR pancreatic] AND [radiomic OR [quantitative AND imaging] OR [texture AND analysis]]. Analysis included cohort size, CT protocol used, radiomic feature (RF) extraction, segmentation, and selection, software used, outcome correlation, and statistical methodology, with focus on reproducibility.

**Results:**

Initial search yielded 1112 articles; however, only 12 articles met all inclusion/exclusion criteria. Cohort sizes ranged from 37 to 352 (median = 106, mean = 155.8). CT slice thickness varied among studies (4 using ≤ 1 mm, 5 using > 1 to 3 mm, 2 using > 3 to 5 mm, 1 not specifying). CT protocol varied (5 using a single portal-venous (pv)-phase, 5 using a pancreas protocol, 1 study using a non-contrast protocol). RF extraction and segmentation were heterogeneous (RF extraction: 5 using pv-phase, 2 using late arterial, 4 using multi-phase, 1 using non-contrast phase; RF selection: 3 pre-selected, 9 software-selected). 2D/3D RF segmentation was diverse (2D in 6, 3D in 4, 2D and 3D in 2 studies). Six different radiomics software were used. Research questions and cohort characteristics varied, ultimately leading to non-comparable outcome results.

**Conclusion:**

The current twelve published IBSI-compliant PDAC radiomic studies show high variability and often incomplete methodology resulting in low robustness and reproducibility.

**Clinical relevance statement:**

Radiomics research requires IBSI compliance, data harmonisation, and reproducible feature extraction methods for non-invasive imaging biomarker discoveries to be valid. This will ensure a successful clinical implementation and ultimately an improvement of patient outcomes as part of precision and personalised medicine.

**Key Points:**

• *Current state of radiomics research in pancreatic cancer shows low software compliance to the Image Biomarker Standardisation Initiative (IBSI).*

• *IBSI-compliant radiomics studies in pancreatic cancer are heterogeneous and not comparable, and the majority of study designs showed low reproducibility.*

• *Improved methodology and standardisation of practice in the emerging field of radiomics has the potential of this non-invasive imaging biomarker in the management of pancreatic cancer.*

**Supplementary Information:**

The online version contains supplementary material available at 10.1007/s00330-023-09653-6.

## Introduction

Pancreatic ductal adenocarcinoma (PDAC) is a malignancy with poor prognosis, frequently presenting at an advanced stage, with a 5-year survival rate of only 11% [1, 2]. Despite advancements in diagnostic and staging technologies such as CT and MR, gains in detection and outcomes have been minimal over the last decades, highlighting the need for more effective and optimised PDAC management.

Radiomics, a novel and promising higher computational method, involves extracting so-called radiomic features (RFs) from medical images that are not discernible by the human eye. The discovery of non-invasive RF-based imaging biomarkers could potentially enable better staging, and lead to improved response to treatment and overall survival as part of precision/personalised medicine [3–5].

The extraction of RFs from regions of interest (ROIs) in two (2D) or three dimensions (3D) is common practice in image analysis. RFs can be broadly classified into first, second, and higher order features: First-order features include shape/sphericity, voxel grey intensity, and coarse voxel distribution which can be represented in a histogram that demonstrates skewness, kurtosis, uniformity, and entropy. Second-order features describe the intensity relationships between neighbouring voxels and include characteristics such as grey-level co-occurrence matrix (GLCM) and grey-level run length matrix (GLRLM) [3, 6]. Lastly, higher order features are extracted through mathematical modulation, or filtering techniques, with the goal of supressing noise or highlighting details and patterns [7].

Despite this vast scope, compared to other solid organ cancers, such as the liver and lungs, PDAC radiomics implementation into clinical practice has been limited [8–10]. This is due to a number of challenges, including difficulties in developing a reliable study design. As shown by Yamashita et al [11], the reproducibility of radiomic models is highly impacted by variations in scanning parameters, such as scanner model used, pixel spacing, and the contrast administration rate, as well as the manual segmentation of ROIs.

Additionally, there are shortcomings in research methodologies, such as data harmonisation and the use of software that adheres to the Image Biomarker Standardisation Initiative (IBSI) guidelines, within an already-complex clinical environment [7, 12, 13].

This review aims to provide an overview of the current state of primary research in PDAC diagnosis, treatment and prognosis, with a particular focus on radiomics and applied methodology. It highlights areas of strength and weakness in the field, with an emphasis on reproducibility and offers guidance to radiomic researchers to generate more robust results.

## Material and methods

### Database search (MEDLINE, PubMed, and Scopus)

A literature search of the online databases MEDLINE, PubMed, and Scopus was conducted between June and August 2022. The search formula contained [pancreas OR pancreatic] AND [radiomic OR [quantitative AND imaging] OR [texture AND analysis]].

Titles and abstracts of articles were initially screened by two raters (H.S.K (radiology subspecialist with 16 years abdominal specialty experience), J.A.M (3rd year medical graduate student)). Inclusion criteria included primary human research articles on CT radiomics in PDAC diagnosis, treatment, and/or prognosis published in English between 2017 and August 2022, and studies using non IBSI-compliant software were excluded. Final selection of articles were then analysed by three raters (H.S.K, J.A.M, M.T. (biostatistician)) (Table 1).

**Table 1:** Inclusion and exclusion assessment criteria performed after initial MEDLINE, PubMed, and Scopus literature search (June–August 2022)

Inclusion criteria	Exclusion criteria
• Literature from 2017 to Aug 2022 • Written in the English language • Primary human research • Relating to PDAC radiomic analysis in the context of diagnosis, treatment, and prognosis • Full text articles (open and non-open access)	• Not relating to PDAC in the context of diagnosis, treatment, and prognosis • Not CT imaging • Not radiomics • Analysis using non-IBSI compliant software

Search keywords: [pancreas OR pancreatic] AND [radiomic OR [quantitative AND imaging] OR [texture AND analysis]]

### Data extraction

Articles were evaluated and categorised according to CT slice thickness (≤ 1 mm, > 1 to 3 mm, > 3 to 5 mm) so to align with prevalent standards and enable comparison of future radiomic investigations. Studies were further subcategorised into various RF clinical applications to address the following research questions: (1) Are there commonly identified RFs across PDAC studies that suggest trends in development of a validated imaging biomarker? (2) What is the reported cohort size, CT technical factors, and described radiomics methodology steps? Are there factors in methods that could impede reproducibility?

Our analysis did not to apply the radiomics quality score (RQS) by Lambin et al [14] given its complex structure (16 components, six key domains, score of 0–36 points) and also based on a recent extensive systematic review comprising 77 articles in high ranking medical journals that demonstrated an overall basic low RQS adherence rate at 38.7%, with many RQS components receiving a score of 0 points [15].

## Results

**Fig. 1:**
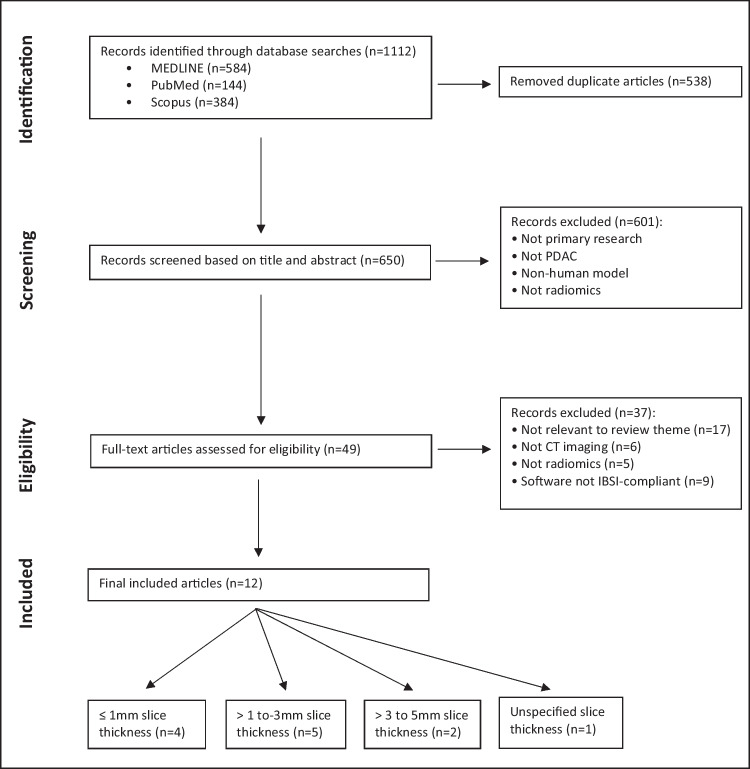
PRISMA flow diagram of MEDLINE, PubMed, and Scopus literature search. Abbreviations: IBSI, Image Biomarker Standardisation Initiative; PDAC, pancreatic ductal adenocarcinoma

Figure 1 illustrates the PRISMA flow diagram outlining the literature search. A total of 1112 articles were found (MEDLINE *n* = 584, PubMed *n* = 144, Scopus *n* = 384), with duplicates removed resulting in a total of 650 articles. The initial screening of titles and abstracts identified 49 articles that met the eligibility criteria for full-text assessment. After further exclusions, a total of 12 articles were included in this review (Table 2).

**Table 2:** Included full-text articles on radiomics in PDAC in this review (in alphabetical order)

Author (year) [reference]	Publishing journal	Aim	Patient cohort size (subcohorts)	CT scan dates (year)	Selected CT contrast phase for RF extraction	CT pancreas protocol	Segmentation dimension	CT slice thickness	Software	Radiomic features (RF)	General comments on methodology
Attiyeh et al (2019) [22]	Annals of Surgical Oncology	OS in resectable PDAC, modelled with CA19-9	161 (113 training, 48 validation)	2009 to 2012	Multiphase	Yes	3D	2.5 mm	MATLAB	Not specified	Selected CT phase not disclosed Significant RFs not disclosed Significance for feature selection defined as *p* < 0.1
Cai et al (2020) [27]	European Radiology	DFS and OS after resection	312 (212 training, 100 validation)	2010 to 2017	Multiphase	Yes	3D	Unknown	MATLAB	Attenuation at tumour/parenchyma interface	No secondary RFs utilised
Chang et al (2020) [24]	Quantitative Imagine in Medicine and Surgery	Differentiate low vs. high grade	301 (151 training, 150 validation)	2005 to 2018	Late arterial	No	2D + 3D	1–3 mm	IBEX	10 RFs, homogeneity, entropy, inverse variance, max probability, information measure correlation, intensity	Extremely heterogeneous cohort RF selection details not disclosed
Chen et al (2021) [16]	Clinical Radiology	SMV and PV invasion	54	2008 to 2018	Portal venous	Unknown	2D	1 mm	AnalysisKit	3 RF RLM parameters	Small cohort Moderate inter-reader variability (κ = 0.517)
Cozzi et al (2019) [21]	PLoS One	OS following SBRT	100 (60 training, 40 validation)	Not specified	Non contrast	No	2D + 3D	2 mm	LIFEx	1 RF GRLNU, 1 RF GLCM homogeneity	CT non-contrast likely inappropriate for radiomics segmentation
Gregucci et al (2022) [18]	Radiologia Medica	PFS and OS following SBRT	37	2014 to 2016	Late arterial	Yes	3D	3 mm	IBEX	Intensity histogram, 1 RF GLCM, 1 RF NID	Small cohort
Hang et al (2021) [17]	Journal of Cancer	OS in patients with liver metastases	39	2016 to 2019	Portal venous	Yes	2D	1 mm	LIFEx	2 RF GLZLM of primary, kurtosis of primary, NGLDM (busyness) of liver metastases	Small cohort RF selection details not disclosed
Healy et al (2022) [19]	European Radiology	Pre-operative model for OS	567 (352 training, 215 validation)	2005 to 2018	Portal venous	No	2D	1 mm	PyRadiomics	First-order features, median and minimum GLCM entropy	Extremely heterogeneous cohort Data harmonisation (original CT slice thickness not disclosed)
Khalvati et al (2019) [26]	Nature	OS in resectable PDAC	98 (30 training, 68 validation)	2007 to 2013	Portal venous	Yes	3D	5 mm (+ 2 mm)	PyRadiomics	GLCM entropy, square root GLCM cluster tendency	Small training cohort Unusual cohort size ratio of training and validation groups RF selection details not disclosed Complex statistical methods (detailed explanation, not disclosed)
Salinas-Miranda et al (2020) [20]	Canadian Association of Radiologists Journal	OS and TTP in unresectable PDAC	112	2015 to 2018	Portal venous	No	2D	1 mm	PyRadiomics	Square root—cluster tendency	Heterogeneous cohort Extremely heterogeneous cohort Data harmonisation (original CT slice thickness not disclosed)
Shi et al (2021) [25]	Pancreatology	OS after upfront surgery	299 (210 training, 89 validation)	2014 to 2017	Multiphase	No	2D	5 mm	Artificial Intelligent Kit	7 RF GLRLM, 3 RF GLCM, 3 RF histogram	Complex statistical methods (detailed explanation, not disclosed)
Tikhonova et al (2022) [23]	European Radiology	Grading according to WHO class	99 (11 grade 1, 51 grade 2, 29 grade 3)	2017 to 2019	Multiphase	Unknown	2D	1.5 mm	LIFEx	GLZLM, GLCM correlation, discretised HU	Very small segmentation volume of interest (1 mm^3^) to assess enhancement changes irrespective of PDAC size

*Abbreviations*: *DFS* disease-free survival, *GLCM* grey-level co-occurrence matrix, *GLRLM* grey-level run length matrix, *GLZLM* grey-level zone length matrix, *GRLNU* grey-level non-uniformity, *NGLDM* neighbourhood grey-level different matrix, *NID* neighbourhood intensity difference, *OS* overall survival, *PFS* progression-free survival, *PV* portal venous, *RF* radiomic feature, *RLM* run length matrix, *SBRT* stereotactic body radiation therapy, *SMV* superior mesenteric vein, *TTP* time to progression

Full-text analysis revealed a lack of common RFs, highly variable methodologies, and a lack of sufficient information to ensure reproducibility (Table 3).

**Table 3:** Missing detailed methodology steps impeding reproducibility

Methodology step	Factor	Author (year) [reference]
Data harmonisation	CT slice thickness not disclosed	Cai et al (2020) [27]
RF selection	General selected RFs not disclosed	Attiyeh et al (2019) [22] Chang et al (2020) [24]
Statistical analysis	Significant threshold for selected RFs *p* < 0.1	Attiyeh et al (2019) [22] Gregucci et al (2022) [18]
Model prediction calculation	Specific selected RFs not disclosed	Attiyeh et al (2019) [22] Gregucci et al (2022) [18] Hang et al (2021) [17] Salinas-Miranda et al (2020) [20]
Model prediction calculation	Multivariable calculation (1- or 2-way) not disclosed	Attiyeh et al (2019) [22] Cai et al (2020) [27] Chang et al (2020) [24] Cozzi et al (2019) [21] Gregucci et al (2022) [18] Hang et al (2021) [17] Healy et al (2022) [19] Khalvati et al (2019) [26] Salinas-Miranda et al (2020) [20] Shi et al (2021) [25] Tikhonova et al (2022) [23]
Model prediction calculation	Handling of missing data not disclosed	Attiyeh et al (2019) [22] Cai et al (2020) [27] Chang et al (2020) [24] Chen et al (2021) [16] Cozzi et al (2019) [21] Gregucci et al (2022) [18] Hang et al (2021) [17] Khalvati et al (2019) [26] Shi et al. (2021) [25] Tikhonova et al (2022) [23]

*RF* radiomic feature

### Cohort size and CT technique

Patient cohort sizes ranged from 37 to 352 (median = 106, mean = 155.8). Three of the 12 selected studies for review had a small cohort size ranging from 37 to 54 [16–18].

Four studies showed a CT slice thickness of ≤ 1 mm [16, 17, 19, 20], five a thickness of > 1–3 mm [18, 21–24], two a thickness of > 3 to 5 mm [25, 26], and one had no slice thickness specified [27] (Table 3). Nine of the 12 studies used single-centre data [16–18, 20–25], 2 studies used data from 2 different centres (one each as a training cohort, one each a validation cohort) [26, 27], and one study used data from 5 centres [19].

A CT pancreas protocol was utilised in 5 out of 12 studies [17, 18, 22, 26, 27], while no pancreas protocol was applied in 5 out of 12 studies [19–21, 24, 25], and 2 studies did not provide information on whether patients underwent a CT pancreas protocol [16, 23]. CT contrast phase used for segmentation varied among studies, with five studies using the pv-phase [16, 17, 19, 20, 26], two studies using the late arterial phase [18, 24], four studies using multiple phases [22, 23, 25, 27], and one study using no contrast agent [21].

### Radiomic feature extraction and selection

RF selection and analysis methodologies were reproducible, albeit highly variable. In three studies, RFs were chosen a priori [18, 20, 23], while the remaining nine studies extracted features from the respective software libraries. Software used was PyRadiomics [19, 20, 26] and LIFEx in three [17, 21, 23], IBEX [18, 24] and MATLAB in two [22, 27], and AnalysisKit [16] and Artificial Intelligent Kit in one study [25].

### Statistical analysis, and model development

Analysed outcome variables applied either time-to-event endpoints modelled using Cox-regression techniques [17–22, 25, 26] or logistic regression models for assessment of PDAC grades [23, 24], local response [18, 21], or superior mesenteric-portal vein invasion [16].

When selecting RFs as part of their models, two studies included RFs as part of their multivariable analysis with *p*-values of < 0.1 for significance [17, 22], while the remaining studies either did not explicitly state any variations, or it was assumed that *p* < 0.05 was considered the threshold. Two studies did not disclose details of the specific RFs chosen [22, 24]. Additionally, one of these studies did not disclose the CT phase associated with the chosen features [22].

Many studies provided details of correlation analysis to reduce the number of features considered in univariable or multivariable modelling [16, 17, 20, 25, 27] and to assess for collinearity between variables [18, 28] upon multivariable modelling. Of the 12 studies, half used least absolute shrinkage and selection operator (LASSO) techniques [19, 23–27]. Step-wise backwards screening methods for statistically significant variable selection was used by one study [16], while elastic net regularisation was also considered [21]. A few studies did not explicitly provide descriptions of the methods of the variable selection in the multivariable prediction model analysis [17, 18, 20, 22]. Only one study indicated the application of a two-way interaction calculation [19].

A combination of receiver operating curve (ROC) or the Harrell’s C-concordance index analysis was applied in all but two studies [20, 26]. Validation and calibration statistical parameters such as Akaike information criteria and calibration curves and/or Hosmer–Lemeshow tests were utilised to evaluate model performance and fit in most studies, with three exceptions [18, 20, 26]. Several papers specified the consideration of categorical feature variables via dichotomisation based on medians [19], Youden index from ROC analysis [24], or other criteria [21].

Half of the reviewed studies [16, 19, 23, 25–27] reported conducting an assessment of inter-observer reliability on the selected RFs for analysis prior to consideration of multivariable modelling.

Two studies detailed the handling of missing data in their modelling analysis [19, 20], and commented on the reasons why this was undertaken.

Three studies acknowledged that internal validation cohorts were not considered due to insufficient sample size [16, 18, 23].

R statistical software was used for all selected studies except for one which utilised SPSS [24].

## Discussion

Radiomics is a computational method of extracting features (RF) from medical images that has the potential to develop non-invasive imaging biomarkers aiding in improved PDAC delineation and treatment. To date, there is limited PDAC CT radiomics primary research data available.

Our analysis revealed several challenges associated with the use of retrospective data from heterogeneous small- to moderately sized cohorts. Furthermore, there was a lack agreement of RFs deemed significant. Additionally, variations in CT techniques and lack of consistency in RF segmentation and selection further hindered reproducibility of findings. However, it is noteworthy that GLCM-associated RFs were observed in 6 of the 12 studies reviewed, albeit without any consistent subcategories.

The median cohort size was 106 patients, with 7 out of the 12 studies having such small cohort sizes rendering a validation process impossible. Training-validation-ratio of cohorts is an important factor in ensuring a robust prognostic model. Training-validation-ratios of medical studies usually range from 67:33 to 80:20 [29]. The study by Khalvati et al [26] demonstrated a training:validation ratio of 30:68 which would necessitate caution when interpreting results. Further statistical considerations include the overfitting of a complex radiomic model and the limitation of Bonferroni corrections which are not applicable when the sample size is too small [18]. Overfitting can be somewhat mitigated by pre-selecting validated RFs; however, demonstration of sufficient power to develop a prognostic model and proper validated studies is scarce as shown in this study.

Another important factor to consider in radiomic studies is variation in CT image acquisition. CT scanning and scanner parameters play a significant role in PDAC staging and influence the performance of radiomic models [11]. Utilising a standardised CT protocol, particularly one that includes a late arterial and portal venous phase as described by the NCCN criteria, would render studies more comparable [30–32]. Five of the 12 studies noted the use of a CT pancreas protocol while 6 out of the 7 remaining studies did not specify the reasoning or acknowledge this limitation. Healy et al [19] did not capture CT scanning protocol variances aiming to develop and validate their radiomics model under “real-world circumstances”. This bears the risk of RF extraction from varied, heterogeneous datasets that may compromise the interpretation of results. As proposed by Noda et al [33], utilising a single portal-venous dual-energy computed tomography image in lieu of a specific CT pancreas protocol may serve as potential solution to standardise image acquisition for radiomics while still maintaining the CT pancreas protocol as gold standard for PDAC staging.

CT technique can greatly impact image quality, and tumour and vessel conspicuity, and ultimately affect radiomics analysis. Studies such as He et al [34] have demonstrated that factors such as contrast enhancement, slice thickness, and convolution kernel reconstruction impact on performance of radiomics models. In order to ensure reproducibility in radiomics studies, data harmonisation is a crucial step. This can include image resampling to maintain a consistent slice thickness, voxel size, and pixel grey intensity ranges (grey-level discretisation) [35]. However, data harmonisation is commonly overlooked, as demonstrated in studies such as Healy et al [19], where a large and heterogeneous cohort was used with multiple CT scanner types and scanning protocols over a larger period of time during which CT scanning imaging techniques were rapidly evolving. The authors state that CT imaging data was harmonised to 1-mm-slice thickness without specifying the original imaging slice thickness data. Given that patients in this cohort were recruited from as early as 2005, it appears unlikely that the original CT slice thickness was ≤ 1 mm, implying that the data was likely not harmonised as intended (i.e., reformatting a 5-mm slice thickness into 5 × 1-mm slice thicknesses). Other studies, such as those by Salinas-Miranda et al [20] and Cai et al [27] have used the same patient cohort with similar harmonisation methods, thus failing to report CT slice thickness. Furthermore, the study by Khalvati et al [26] used different CT slice thicknesses in their training and validation cohorts (5 mm and 2 mm respectively) without acknowledging the impact on radiomic analysis.

A recent endeavour to streamline radiomics analysis and to enhance RF reproducibility is the IBSI [12]. The 160 + pages framework places a significant emphasis on mathematical and technical aspects, while giving less attention to the clinical implementation. This is likely due to the fact that the majority of authors are of non-clinical backgrounds. As a result, clinicians have to trust and rely on IBSI-compliant radiomics software for quality assurance and reproducibility purposes. This is also underpinned in a study by Fornacon-Wood et al, which found a higher number of RFs exhibiting excellent statistical reliability when extracted using IBSI-compliant software, as opposed to non-compliant software [13]. In our initial eligibility assessment, we identified 9 of 49 studies that used non-IBSI compliant software, and as a result, these studies were excluded from our final analysis.

Statistical extrapolations and radiomic models were noted to be highly variable. For instance, Hang et al [17] sought to correlate RFs of primary PDAC tumours and liver metastases to overall survival by incorporating four texture features into a radiomics score based on a statistical significance of *p* < 0.1, as opposed to the commonly employed threshold of *p* < 0.05. Furthermore, the authors failed to provide information on whether selected RFs were considered for or actually included in the multivariable model. Similarly, Attiyeh et al [22] developed two models to predict overall survival in resectable PDAC patients, incorporating additional characteristics such as serum CA19-9 and Brennan pathology scoring. However, the chosen CT contrast phase for RF extraction as well as RF selection was not disclosed for either model or univariate analysis. Two studies that aimed to use radiomics to assess tumour grading are limited by their methodologies and reporting [23, 24]. Chang et al [24] did not disclose details regarding the significant RF selection while Tikhonova et al [23] used a *p*-value of < 0.1 for statistical significance and assessed contrast enhancement changes in a very small volume of interest (< 1mm^3^).

The study by Chen et al [16] showed the potential utility of RFs in identifying and correlating suspected superior mesenteric vein and suspected portal vein invasion. Their model showed superior performance in comparison to two experienced radiologists. Despite the limitations (small cohort (*n* = 54) and moderate inter-reader variability (κ = 0.517)), this model exemplifies a robust, IBSI compliant methodology that warrants future validation.

Modelling techniques, such as the LASSO algorithm (least absolute shrinkage and selection operator), are useful in identifying significant variables and features of data. However, our analysis showed that many studies, if not all, may be underpowered, resulting in an inability to detect statistically significant clinically prognostic features. Despite acknowledging the limitation of small sample size, many studies suggest that validating the identified final model using an internal or external validation dataset is sufficient, as opposed to reproducing the model with a larger, adequately powered sample size to identify statistically significant features. The predictive performance of models is likely to benefit from an assessment of interactions between variables, but only the study by Hang et al with the largest sample size (*n* = 352) could feasibly allow for this exploration.

Study reviewed did not provide a link to the data, statistical analysis, or programming code used, which might be due to patient data confidentiality reasons. While the majority of studies were transparent in their methods, several omissions were noted which impede reproducibility.

Recruitment of large PDAC cohort, extracting robust RFs, and developing an imaging biomarker from a potential pool of thousands of RFs with such small sample sizes is challenging. The development of effective methodologies and early engagement of a multidisciplinary team, including more technical, non-clinical craft groups, such as biostatisticians and computer scientists, would greatly benefit research in this field.

## Conclusion

There is a limited number of primary research publications of PDAC CT radiomics using IBSI compliant software. However, as advancements in methodology and standardisation of practice continue to develop, radiomics has the potential to serve as a valuable non-invasive biomarker in the management of pancreatic cancer.

### Supplementary Information

Below is the link to the electronic supplementary material.

Supplementary file1 (PDF 108 KB)

## Abbreviations

2D: Two dimensions
3D: Three dimensions
CT: Computed tomography
DECT: Dual-energy computed tomography
DFS: Disease-free survival
GLCM: Grey-level co-occurrence matrix
GLRLM: Grey-level run length matrix
GLZLM: Grey-level zone length matrix
GRLNU: Grey-level non-uniformity
IBSI: Image Biomarker Standardisation Initiative
LASSO: Least absolute shrinkage and selection operator
NGLDM: Neighbourhood grey-level different matrix
NID: Neighbourhood intensity difference
OS: Overall survival
PDAC: Pancreatic ductal adenocarcinoma
PFS: Progression-free survival
PV: Portal venous
RF: Radiomic feature
RLM: Run length matrix
ROC: Receiver operating curve
ROI: Region of interest
RQS: Radiomics quality score
SBRT: Stereotactic body radiation therapy
SMV: Superior mesenteric vein
TTP: Time to progression

## Declarations

### Guarantor

The scientific guarantor of this publication is Dr. Hyun Soo Ko.

### Statistics and biometry

One of the authors is a licenced academic biostatistician.

### Study subjects or cohorts overlap

NA

### Methodology

• retrospective

• observational

• systematic analysis performed at one institution

## References

[1] Rawla P, Sunkara T, Gaduputi V (2019). Epidemiology of pancreatic cancer: global trends, etiology and risk factors. World J Oncol.

[2] Siegel RL, Miller KD, Fuchs HE, Jemal A (2022). Cancer statistics, 2022. CA Cancer J Clin.

[3] Marti-Bonmati L, Cerdá-Alberich L, Pérez-Girbés A et al (2022) Pancreatic cancer, radiomics and artificial intelligence. Br J Radiol 95(1137):20220072 10.1259/bjr.20220072 PMC10996946 35687700

[4] Abunahel BM, Pontre B, Kumar H, Petrov MS (2021). Pancreas image mining: a systematic review of radiomics. Eur Radiol.

[5] Casà C, Piras A, D’Aviero A et al (2022) The impact of radiomics in diagnosis and staging of pancreatic cancer. Ther Adv Gastrointest Endosc 15:26317745221081596 10.1177/26317745221081596 PMC8943316 35342883

[6] Bartoli M, Barat M, Dohan A (2020). CT and MRI of pancreatic tumors: an update in the era of radiomics. Jpn J Radiol.

[7] Rizzo S, Botta F, Raimondi S et al (2018) Radiomics: the facts and the challenges of image analysis. Eur Radiol Exp 2(1):36 10.1186/s41747-018-0068-z PMC6234198 30426318

[8] Bibault JE, Xing L, Giraud P et al (2020) Radiomics: a primer for the radiation oncologist. Cancer Radiother 24(5):403–410 10.1016/j.canrad.2020.01.011 32265157

[9] Park HJ, Park B, Lee SS (2020). Radiomics and deep learning: hepatic applications. Korean J Radiol.

[10] Yang F, Zhang J, Zhou L et al (2022) CT-based radiomics signatures can predict the tumor response of non-small cell lung cancer patients treated with first-line chemotherapy and targeted therapy. Eur Radiol 32(3):1538–1547 10.1007/s00330-021-08277-y 34564744

[11] Yamashita R, Perrin T, Chakraborty J, Chou JF, Horvat N, Koszalka MA et al (2020) Radiomic feature reproducibility in contrast-enhanced CT of the pancreas is affected by variabilities in scan parameters and manual segmentation. Eur Radiol 30(1):195–205 10.1007/s00330-019-06381-8 PMC7127865 31392481

[12] Zwanenburg A, Vallieres M, Abdalah MA et al (2020) The image biomarker standardization initiative: standardized quantitative radiomics for high-throughput image-based phenotyping. Radiology 295(2):328–338 10.1148/radiol.2020191145 PMC7193906 32154773

[13] Fornacon-Wood I, Mistry H, Ackermann CJ et al (2020) Reliability and prognostic value of radiomic features are highly dependent on choice of feature extraction platform. Eur Radiol 30(11):6241–6250 10.1007/s00330-020-06957-9 PMC7553896 32483644

[14] Lambin P, Leijenaar RTH, Deist TM, Peerlings J, de Jong EEC, van Timmeren J et al (2017) Radiomics: the bridge between medical imaging and personalized medicine. Nat Rev Clin Oncol 14(12):749–762 10.1038/nrclinonc.2017.141 28975929

[15] Park JE, Kim D, Kim HS et al (2020) Quality of science and reporting of radiomics in oncologic studies: room for improvement according to radiomics quality score and TRIPOD statement. Eur Radiol 30(1):523–536 10.1007/s00330-019-06360-z 31350588

[16] Chen F, Zhou Y, Qi X, Xia W, Zhang R, Zhang J et al (2021) CT texture analysis for the presurgical prediction of superior mesenteric-portal vein invasion in pancreatic ductal adenocarcinoma: comparison with CT imaging features. Clin Radiol 76(5):358–366 10.1016/j.crad.2021.01.003 33581837

[17] Hang J, Xu K, Yin R et al (2021) Role of CT texture features for predicting outcome of pancreatic cancer patients with liver metastases. J Cancer 12(8):2351–2358 10.7150/jca.49569 PMC7974874 33758611

[18] Gregucci F, Fiorentino A, Mazzola R et al (2022) Radiomic analysis to predict local response in locally advanced pancreatic cancer treated with stereotactic body radiation therapy. Radiol Med 127(1):100–107 10.1007/s11547-021-01422-z 34724139

[19] Healy GM, Salinas-Miranda E, Jain R et al (2022) Pre-operative radiomics model for prognostication in resectable pancreatic adenocarcinoma with external validation. Eur Radiol 32(4):2492–2505 10.1007/s00330-021-08314-w 34757450

[20] Salinas-Miranda E, Khalvati F, Namdar K et al (2021) Validation of prognostic radiomic features from resectable pancreatic ductal adenocarcinoma in patients with advanced disease undergoing chemotherapy. Can Assoc Radiol J 72(4):605–613 10.1177/0846537120968782 33151087

[21] Cozzi L, Comito T, Fogliata A et al (2019) Computed tomography based radiomic signature as predictive of survival and local control after stereotactic body radiation therapy in pancreatic carcinoma. PLoS One 14(1):1–11 10.1371/journal.pone.0210758 PMC6338357 30657785

[22] Attiyeh MA, Chakraborty J, Doussot A et al (2018) Survival prediction in pancreatic ductal adenocarcinoma by quantitative computed tomography image analysis. Ann Surg Oncol 25(4):1034–1042 10.1245/s10434-017-6323-3 PMC6752719 29380093

[23] Tikhonova VS, Karmazanovsky GG, Kondratyev EV et al (2022) Radiomics model-based algorithm for preoperative prediction of pancreatic ductal adenocarcinoma grade. Eur Radiol 33:1152–1161 10.1007/s00330-022-09046-1 35986774

[24] Chang N, Cui L, Luo Y, Chang Z, Yu B, Liu Z (2020). Development and multicenter validation of a CT-based radiomics signature for discriminating histological grades of pancreatic ductal adenocarcinoma. Quant Imaging Med Surg.

[25] Shi H, Wei Y, Cheng S et al (2021) Survival prediction after upfront surgery in patients with pancreatic ductal adenocarcinoma: radiomic, clinic-pathologic and body composition analysis. Pancreatology 21(4):731–737 10.1016/j.pan.2021.02.009 33678581

[26] Khalvati F, Zhang Y, Baig S et al (2019) Prognostic value of CT radiomic features in resectable pancreatic ductal adenocarcinoma. Sci Rep 9(1):5449 10.1038/s41598-019-41728-7 PMC6443807 30931954

[27] Cai X, Gao F, Qi Y et al (2020) Pancreatic adenocarcinoma: quantitative CT features are correlated with fibrous stromal fraction and help predict outcome after resection. Eur Radiol 30(9):5158–5169 10.1007/s00330-020-06853-2 32346792

[28] Almeida LS, Teixeira CJ, Campos CV et al (2022) Low birth weight intensifies changes in markers of hepatocarcinogenesis induced by fructose consumption in rats. Metabolites 12(10):886 10.3390/metabo12100886 PMC9608855 36295788

[29] Steyerberg EW (2019) Clinical prediction models: a practical approach to development, validation, and updating / Ewout W. Steyerberg. Second edition. ed: Springer

[30] Al-Hawary M (2016). Role of imaging in diagnosing and staging pancreatic cancer. J Natl Compr Canc Netw.

[31] Tempero MA, Malafa MP, Al-Hawary M et al (2021) Pancreatic adenocarcinoma, Version 2.2021, NCCN Clinical Practice Guidelines in Oncology. J Natl Compr Canc Netw 19(4):439–57 10.6004/jnccn.2021.0017 33845462

[32] Almeida RR, Lo GC, Patino M, Bizzo B, Canellas R, Sahani DV (2018). Advances in pancreatic CT imaging. AJR Am J Roentgenol.

[33] Noda Y, Tochigi T, Parakh A, Joseph E, Hahn PF, Kambadakone A (2021). Low keV portal venous phase as a surrogate for pancreatic phase in a pancreatic protocol dual-energy CT: feasibility, image quality, and lesion conspicuity. Eur Radiol.

[34] He L, Huang Y, Ma Z, Liang C, Liang C, Liu Z (2016). Effects of contrast-enhancement, reconstruction slice thickness and convolution kernel on the diagnostic performance of radiomics signature in solitary pulmonary nodule. Sci Rep.

[35] van Timmeren JE, Cester D, Tanadini-Lang S, Alkadhi H, Baessler B (2020). Radiomics in medical imaging-"how-to" guide and critical reflection. Insights Imaging.

