# Supplementary material for: Current state of radiomic research in pancreatic cancer: focusing on study design and reproducibility of findings
Source: Eur Radiol. 2023 Apr 20;33(10):6659–69. doi: 10.1007/s00330-023-09653-6 (PMC10511615; doi:10.1007/s00330-023-09653-6)
Supplement: Supplementary file 1 — Supplementary file1 (PDF 108 KB) [file 330_2023_9653_MOESM1_ESM.pdf]

ELECTRONIC SUPPLEMENTARY MATERIAL

Current State of Radiomic Research in Pancreatic Cancer: Focusing on Study Design and Reproducibility of Findings

Supplemental Table 1

Author (year) (reference)	Publishing journal	Aim	Patient cohort size	Software	Radiomic features and general comments on methodology
Borhani et al. (2019) [1]	American Journal of Roentgenology	Assessing response to neoadjuvant therapy in terms of CA19-9	39	TexRAD	• Histogram-based: first-order statistics of grey-level histograms<br>• Inclusion and exclusion criteria not very detailed<br>• Data harmonization (CT slice thickness)<br>• Significant p=0.049 delta kurtosis is borderline significant and might become non-significant when data is corrected for above mentioned factors
Ciaravino et al. (2018) [2]	Anticancer Research	Assessing response to neoadjuvant therapy in terms of downgrading to resectable	17	MaZda	• Histogram - mean value, variance, skewness, kurtosis and entropy, obtained pre and post treatment<br>• Small and heterogeneous cohort (resectable/ borderline resectable)
Chen et al. (2017) [3]	PLoS One	Assessing daily RF changes during (neo-) adjuvant chemoradiation therapy (28 fractions)	20	Internally modified Matlab	• Histogram- mean HU, peak position (PP), total voxel volume, SD, skewness, kurtosis, energy, and entropy<br>• Small and heterogeneous cohort (15/20 resected, 5/20 non resected)<br>• Main conclusion is based on a single patient who showed no lower tumour HU density post chemoradiation therapy that correlated with poorer outcome
Kim et al. (2019) [4]	European Radiology	Predicting resectability and overall survival after neoadjuvant therapy	45	Internally developed	• Histogram and second-order texture parameters<br>• Two readers assessed regarding resectability status<br>• Image based deemed borderline resectable cases were re-categorized into resectable or unresectable based on whether R0 resection was deemed feasible (no details on how R0 status was determined)<br>• A third, single reader performed ROI annotation<br>• A fourth, single reader performed RF-extraction based on ROI annotation
Cassinotto et al. (2017) [5]	European Journal of Radiology	Disease free survival in relation to lymph node involvement and tumour grade	99	TexRAD	• Histogram - pixel HU, SD of pixel HU (variation or dispersion from pixel mean HU), entropy, mean of positive pixel HU (pixel mean HU), skewness, kurtosis
Kim et al. (2019) [6]	Nature Scientific reports	Predicting stage, differentiation, perineural invasion, LN status and overall survival	116	Internally developed	• GLRLM analysis<br>• Very small 2D ROI (approx.. 4mm diameter) placed in centre of PDAC primary, therefore likely not meaningful
Cheng et al. (2019) [7]	European Journal of Radiology	Correlating with overall survival and progression-free survival	41	TexRAD	• Histogram- mean HU, entropy, mean of positive pixels, kurtosis, SD of kurtosis, and skewness for fine to coarse textures
Fang et al. (2020) [8]	Cancer Imaging	Predicting nodal involvement	155	MaZda	• ~300 features, histogram, co-occurrence matrix, run-length matrix, image gradient, auto-regressive model and Haar wavelet<br>• Unusual CT slice thickness used (1.5mm and 5mm)

Rigiroli et al. (2021) [9]	Radiology	RF of PDAC primary and perivascular tissue in correlation to superior mesenteric involvement	194	SyngoVia Frontier Radiomics, version 1.2.5; Siemens Healthineers	• 160/ 1695 RFs with high intraclass correlation coefficient ≥0.7.<br>• 11 RFs selected for logistic regression model building and subsequent 6 highly correlating RFs removed, resulting in 5 RFs

Supplemental Table 1: List of nine excluded studies that utilised non-IBSI compliant software.

Abbreviations: CA 19-9- Carbohydrate antigen 19-9, GLRLM – grey-level run length matrix, HU- Hounsfield units, PDAC- pancreatic adenocarcinoma, RF- radiomic feature, ROI- region of interest

Supplemental Table 1 References:

1. Borhani AA, Dewan R, Furlan A, Seiser N, Zureikat AH, Singhi AD, et al. Assessment of Response to Neoadjuvant Therapy Using CT Texture Analysis in Patients With Resectable and Borderline Resectable Pancreatic Ductal Adenocarcinoma. AJR Am J Roentgenol. 2020;214(2):362-9.
2. Ciaravino V, Cardobi N, DE Robertis Rs, Capelli P, Melisi D, Simionato F, et al. CT Texture Analysis of Ductal Adenocarcinoma Downstaged After Chemotherapy. Anticancer Res. 2018;38(8):4889-95.
3. Chen X, Oshima K, Schott D, Wu H, Hall W, Song Y, et al. Assessment of treatment response during chemoradiation therapy for pancreatic cancer based on quantitative radiomic analysis of daily CTs: An exploratory study. PLoS One. 2017;12(6):e0178961.
4. Kim BR, Kim JH, Ahn SJ, Joo I, Choi SY, Park SJ, et al. CT prediction of resectability and prognosis in patients with pancreatic ductal adenocarcinoma after neoadjuvant treatment using image findings and texture analysis. Eur Radiol. 2019;29(1):362-72.
5. Cassinotto C, Chong J, Zogopoulos G, Reinhold C, Chiche L, Lafourcade JP, et al. Resectable pancreatic adenocarcinoma: Role of CT quantitative imaging biomarkers for predicting pathology and patient outcomes. Eur J Radiol. 2017;90:152-8.
6. Kim HS, Kim YJ, Kim KG, Park JS. Preoperative CT texture features predict prognosis after curative resection in pancreatic cancer. Sci Rep. 2019;9(1):17389.
7. Cheng SH, Cheng YJ, Jin ZY, Xue HD. Unresectable pancreatic ductal adenocarcinoma: Role of CT quantitative imaging biomarkers for predicting outcomes of patients treated with chemotherapy. European Journal of Radiology. 2019;113:188-97.
8. Fang WH, Li XD, Zhu H, Miao F, Qian XH, Pan ZL, et al. Resectable pancreatic ductal adenocarcinoma: association between preoperative CT texture features and metastatic nodal involvement. Cancer Imaging. 2020;20(1):17.
9. Rigiroli F, Hoye J, Lerebours R, Lafata KJ, Li C, Meyer M, et al. CT Radiomic Features of Superior Mesenteric Artery Involvement in Pancreatic Ductal Adenocarcinoma: A Pilot Study. Radiology. 2021;301(3):610-22.
